# Inflammasomes during SARS-CoV-2 infection and development of their corresponding inhibitors

**DOI:** 10.3389/fcimb.2023.1218039

**Published:** 2023-06-09

**Authors:** Rominah Onintsoa Diarimalala, Yanhong Wei, Da Hu, Kanghong Hu

**Affiliations:** Sino-German Biomedical Center, National “111” Center for Cellular Regulation and Molecular Pharmaceutics, Cooperative Innovation Center of Industrial Fermentation (Ministry of Education & Hubei Province), Key Laboratory of Fermentation Engineering (Ministry of Education), Hubei University of Technology, Wuhan, China

**Keywords:** SARS-CoV-2, inflammasome, inhibitors, innate immune response, NOD-like receptor family pyrin domain containing 3 (NLRP3)

## Abstract

Corona Virus Disease 2019 (COVID-19) continues to be a burden for human health since its outbreak in Wuhan, China in December 2019. Recently, the emergence of new variants of concerns (VOCs) is challenging for vaccines and drugs efficiency. In severe cases, SARS-CoV-2 provokes inappropriate hyperinflammatory immune responses leading to acute respiratory distress syndrome (ARDS) and even death. This process is regulated by inflammasomes which are activated after binding of the viral spike (S) protein to cellular angiotensin-converting enzyme 2 (ACE2) receptor and triggers innate immune responses. Therefore, the formation of “cytokines storm” leads to tissue damage and organ failure. NOD-like receptor family pyrin domain containing 3 (NLRP3) is the best studied inflammasome known to be activated during SARS-CoV-2 infection. However, some studies suggest that SARS-CoV-2 infection is associated with other inflammasomes as well; such as NLRP1, absent in melanoma-2 (AIM-2), caspase-4 and -8 which were mostly found during dsRNA virus or bacteria infection. Multiple inflammasome inhibitors that exist for other non-infectious diseases have the potential to be used to treat severe SARS-CoV-2 complications. Some of them have showed quite encouraging results during pre- and clinical trials. Nevertheless, further studies are in need for the understanding and targeting of SARS-Cov-2-induced inflammasomes; mostly an update of its role during the new VOCs infection is necessary. Hence, this review highlights all reported inflammasomes involved in SARS-CoV-2 infection and their potential inhibitors including NLRP3- and Gasdermin D (GSDMD)-inhibitors. Further strategies such as immunomodulators and siRNA are also discussed. As highly related to COVID-19 severe cases, developing inflammasome inhibitors holds a promise to treat severe COVID-19 syndrome effectively and reduce mortality.

## Introduction

**Figure 1:**
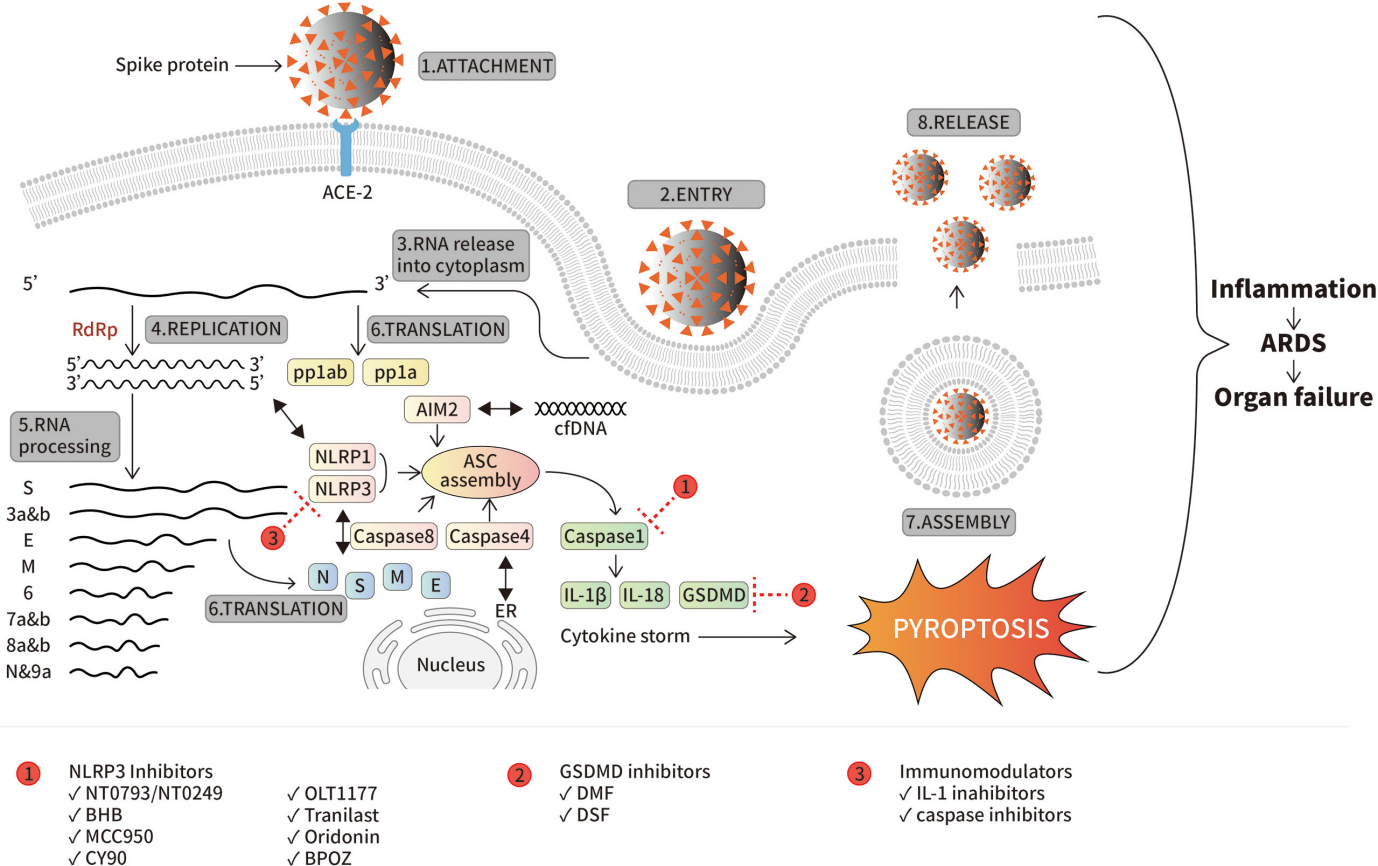
SARS-CoV-2 life cycle and inflammasomes activation. The attachment of the virus by its spike protein to ACE-2 is the first step inducing not only viral entry but also host immune responses. SARS-CoV-2 encodes for multiple ORFs (from S to 9a) by RNA-dependent RNA-polymerase (RdRp). While all structural and non-structural proteins are expressed, viral assembly occurs ending into release of new virions. Meanwhile, inflammasomes are highly activated by innate immune system in order to eliminate viral infection. Mechanistically, NLRP3 is activated by viral N and S protein, NLRP1 by the dsRNA formed during viral life cycle, AIM-2 by cfDNA and caspase-4 and -8. Activation of all those inflammasomes promotes ASC assembly and activation of caspase-1 following by expression of IL-1β, IL-18 and GSDMD (“cytokines storm”) resulting in pyroptosis which is mainly responsible of tissue damage leading to ARDS and organ failure during virus infection. The red circles containing the numbers 1, 2, and 3 represent three types of inflammasome inhibitors, and the positions of their intervening targets are indicated with a red dash line T, respectively.

SARS-CoV-2 is a positive single-stranded RNA (ssRNA) virus belonging to *Betacoronavirus* genus, *coronaviridae* family. It has a large genome of 29.9kb within 13 open reading frames (ORFs). ORF1a and ORF1b permit the primary translation of a polyprotein to initiate infection (Kim et al., 2020). Subsequently, after some replications, the other ORFs assure the expression of the subgenomic mRNA encoding for all structural proteins (Sola et al., 2015). Until now, SARS-CoV-2 is provoking infection resulting in human’s hospitalization and death worldwide. The most people at risk are the ones with advanced age, hypertension, cardiovascular disease and diabetes (Wang et al., 2020). The main targets for antiviral development are RNA-dependent RNA-polymerase (RdRp), viral proteases or ACE-2 cell receptors (Kruse, 2020; Majumder and Minko, 2021). However, their progress in FDA approval is challenging due to their moderate effect and the rapid emergence of new virus variants (Robinson et al., 2022). Similarly, vaccines are being put into use by some commercial companies such as Jonhson & Johnson or Sinovac Biotech but their immune protection efficacy is far from satisfactory due to the fast evolution of SARS-CoV-2 (Hadj Hassine, 2022). COVID-19 symptoms are mild and self-limiting or in many cases asymptomatic. However, some of the infected people may develop severe symptoms which can lead to death (Huang et al., 2020). Indeed, it has infected 700 million people so far with 6.8 million deaths (COVID-19 Map - *Johns Hopkins Coronavirus Resource Center (jhu.edu)*, access on 09.03.2023). Studies have proven that many severe cases are due to inappropriate hyperinflammatory responses of the immune system to the viral infection (Kaivola et al., 2021; Vora et al., 2021). In fact, immune system responses are the first line of defense against viral infection. Once the spike (S) protein of SARS-CoV-2 binds to the cell receptors, pattern-recognition receptors such as Nod-like receptor (NLR) or absent in melanoma-2 (AIM2) will assemble inflammasomes through inducing membrane pore formation and activation of proinflammatory cytokines in order to activate cell death and eliminate the viral infection (**Figure 1**). Indeed, during SARS-CoV-2 infection, those proteins are highly promoted. Therefore, the over-responses induce lung injuries leading to severe symptoms including acute respiratory distress syndrome (ARDS) and subsequent death (Lee et al., 2020). Thus, controlling this response of the immune system is essential and represents a valuable key for the fight against COVID-19 leading to severe symptoms (Yap et al., 2020; Rodrigues et al., 2021). This review highlights the involved inflammasomes during SARS-CoV-2 infection and summarizes their corresponding inhibitors. Consequently, the information presented would be helpful in the understanding of viral pathogenesis mechanism, the understanding of inflammasome roles during SARS-Cov-2 infection and development of novel therapeutic molecules against COVID-19. Moreover, it provides insights on future study direction of the virus as it is quite new and a lot remain unknown.

## Possible inflammasome targets

Excessive inflammatory responses induced upon SARS-CoV-2 infection are associated with severe symptoms of COVID-19. NLRP3 is the most studied inflammasome and the first known to be activated during SARS-CoV-2 infection (Freeman and Swartz, 2020). However, other inflammasomes such as NLRP1, AIM2, caspase-4 and -8 have a quite high possibility of involvement in activating cytokines that induce the over-responses of the immunity and the severity of the disease.

### NOD-, LRR- and pyrin domain-containing protein 3 inflammasome

It is an essential component of the immune system and consists of a sensor (NLRP3), an adaptor (apoptosis-associated speck-like protein containing a caspase recruitment domain, ASC) and an effector (caspase-1). During harmful stimuli such as viral invasion, NLRP-3 induces ASC assembly which recruits caspase-1, resulting to the cleavage of pro-IL-1β and pro-IL-18 into their active forms IL-1β and IL-18. These latter are important cytokines that assure the inflammatory response of the immune system during the viral infection (Kaivola et al., 2021). However, in some cases, NLRP3 response is associated with serious diseases like autoinflammatory disease. Recently, it was proven to provoke severe symptoms in COVID-19 patients (Zhao et al., 2021). Furthermore, study in macrophages and dentritic cells as well as mice model revealed the promotion of mature cytokines by viral N protein which led to lung injuries. Mechanistically, N protein interacts directly with NLRP3 complex, promotes the binding of NLRP3 with ASC, and facilitates NLRP3 inflammasome assembly (Pan et al., 2021). Indeed, autopsy of COVID-19 patients revealed the presence of active NLRP3 in their peripheral blood mononuclear cells (PBMCs) and tissues (He et al., 2006). Moreover, in patients with severe symptoms, abundant of inflammasome-derived products, such as caspase-1, IL-6, IL-18 and LDH, were found in their sera (Rodrigues et al., 2021). The activation of NLRP3 during COVID-19 infection is via ACE-2 which is highly expressed at the monocyte of COVID-19 patients only (Junqueira et al., 2021). The ssRNA sequences of SARS-CoV-2 trigger activation of important immune molecules, such as TLRs or RIG-1, NF-*K*B promoting production of IL-1β, IL-18, LDH release, and mostly caspase-1 activation as the hallmark of inflammasome assembly which results in GSDMD activation followed by pyroptosis leading to tissue damage and ARDS (Campbell et al., 2021) (**Figure 1**). Furthermore, NLRP3 activation requires efflux of potassium and ASC (Xu et al., 2022). Thus, NLRP3 inflammasome represents an attractive target in the development of drugs for COVID-19 patients with severe manifestations.

### NOD-, LRR- and pyrin domain-containing protein 1 inflammasome

Instead of NLRP3, this inflammasome pathway is initiated by NLRP1 as sensor. The mechanism involved seems to be the same as that for NLRP3 in which the cleavage of caspase-1 leads to IL-1β and IL-18 activation and pyroptosis (Bauernfried et al., 2021). Screening of different types of viruses for inflammasome responses in keratinocytes revealed that NLRP1 acts as a biosensor for double-stranded RNA (dsRNA) virus (Bauernfried et al., 2021). Indeed, a negative-strand RNA is synthesized by SARS-CoV-2 to serve as template for progeny viral genome production, this process is similar to other coronaviruses life cycle (Sola et al., 2015). Therefore, it is highly possible that viral dsRNA activates NLRP1 by binding to it through a leucine-rich repeat domain of NLRP1 and participates together with NLRP3 to the over-responses of the immune system leading to COVID-19 severe symptoms (Bauernfried et al., 2021). Moreover, viral proteases like EV-A71 3C^pro^ are able to activate NLRP1 in some cases through direct cleavage (Robinson et al., 2020). Therefore, SARS-CoV-2 proteases (PL2 and 3CL^pro^) are likely to be involved in the activation of NLRP1 inflammasome (Planès et al., 2022).

### Absent in melanoma 2 inflammasome

AIM2 is the third sensor that is related to inflammasome activation during infection (Kaivola et al., 2021). It is associated predominantly to dsRNA virus or bacteria (Lugrin and Martinon, 2018). The mechanism is through ASC assembly and activation of caspase-1 resulting in cytokines expression and pyroptosis. Despite being a ssRNA virus, SARS-CoV-2 was associated with AIM-2 during its study in monocytes. Indeed, considerable levels of AIM-2 were detected in monocytes infected by SARS-CoV-2 (Junqueira et al., 2021). Furthermore, AIM-2 receptor activation was observed in peripheral blood mononuclear cells (PBMCs) from post-Covid-19 patients that presented lung injuries (Colarusso et al., 2022). It was speculated that the ability of SARS-CoV-2 to induce AIM2 inflammasome response is due to the presence of cell free DNA (cfDNA) in several tissues of COVID-19 patient including lungs. In fact, high level of cfDNA was found in COVID-19 patients that required intensive treatment or died during hospitalization (Andargie et al., 2021). Therefore, AIM2 represents a valuable marker and/or target for COVID-19 disease severity and corresponding treatment.

### Caspase-4

Caspase-4 is a protein that participates to the inflammatory response during pathogen invasion (Viganò et al., 2015). *Kaivola et al. s*peculated the involvement of the non-canonical inflammasome caspase-4 in COVID-19 inflammatory response due to both caspase-4 and SARS-CoV-2 localization on the endoplasmic reticulum (ER) membrane (Kaivola et al., 2021). Interestingly, recent studies confirmed that the caspase-4 and its homolog in mice, caspase 11, were associated with COVID-19 disease severity (Eltobgy et al., 2022). Nevertheless, the exact role of caspase-4 in inducing inflammation during viral infection remains unclear and needs further investigation.

### Caspase-8

It is another caspase that induce inflammation during SARS-CoV-2 infection. What is known so far about its involvement in SARS-CoV-2 infection is its activation. In fact, SARS-CoV-2 triggered caspase-8 activation, expression of IL-1β and IL-18 resulting in apoptosis in lung epithelial cells (Li et al., 2020).

## SARS-Cov-2 variants and inflammasomes

Multiple variants of SARS-Cov-2 have been emerging since its first apparition in Wuhan at the end of 2019. The virus has adapted quickly to humans and became highly mutated mostly at its spike protein sequence which is responsible of the attachment of the virus to ACE-2 cell surface receptor (Awadasseid et al., 2021; Harvey et al., 2021). To date, 5 of the existing variants are considered VOCs or variant of concerns by World Health Organization (WHO). VOCs have a high transmission rate compared to others: rapid spread and quick circulating dominance. In fact, most times, when a new variant emerges, the old one is no longer circulating. They are Alpha, Beta, Gamma and O’micron variants (Scovino et al., 2022). Their direct relation on how they handle immune responses including inflammasome is not yet elucidated. Through early studies on this new virus, inflammasome was positively correlated to lead the viral infection into severe disease by over-secreting cytokines and thus provoke tissue damages and reduce clinical outcomes (**Figure 1**) (Li et al., 2020; Junqueira et al., 2021; Kaivola et al., 2021; Vora et al., 2021). In fact, each variant has different transmission rate and pathogenicity (Kumar et al., 2022). It may be due to cross-protections acquired during past infection or for people that were vaccinated. Interestingly, studied showed that vaccinated people with the first strain Wuhan-hu have protection against Alpha, Beta and Gamma variant but not for O’micron which has more than 15 mutations at the spike receptor binding domain (RBD) plus multiple deletions and substitutions in the N-terminal (Cameroni et al., 2022; Zeng et al., 2022). Furthermore, a study in UK showed that there was a T cell strong memory in patients infected with SARS-Cov-2. Concerning the inflammasome response to SARS-Cov-2 new variants, deeper studies are in need to further confirm the statement of previous studies that obviously showed correlation of inflammasomes and severe COVID-19 (Peng et al., 2020). Hence, investigating the relationship between inflammasomes and the different VOCs will ultimately give more understanding on how the organism reacts towards the infection and how to limit occurrence of severe cases. Furthermore, NLRP3 inflammasome which is the most related to SARS-Cov-2 has different variants as well. Study proved that NLRP3 variants are also responsible of different responses and then clinical symptoms: severe or mild. Indeed, NLRP3 rs10157379 and rs10754558 are the ones positively related to severe COVID-19 (Maes et al., 2022).

## Drugs developments and strategies targeting a certain type of inflammasomes

As a major class of anti-inflammatory molecules, series of compounds targeting a certain type of inflammasome are currently in the preclinical and clinical stages, and their indications include some non-infectious diseases such as atherosclerosis, Alzheimer, Parkinson, gout, rheumatoid arthritis and non-alcoholic steatohepatitis (Awadasseid et al., 2021; Harvey et al., 2021). For infectious diseases like SARS-CoV-2 infection, people may be worried whether the blockage of IL-1 increases the risk of infection. After all, cytokines are natural protein that the organism needs to protect against pathogens. However, the risk shall be reduced as there are multiple ways that innate immune response use to produce IL-1 during infection. Therefore, it makes NLRP3 the most attractive inflammasome to target (Awadasseid et al., 2021; Harvey et al., 2021). To date, there are no commercialized inflammasome inhibitors specifically applied for the treatment of SARS-CoV-2 infection (Declercq et al., 2022). Nevertheless, several existing ones for other diseases are being tested for COVID-19. The results seem encouraging, which brings a glimmer of hope for conquering COVID-19 induced ARDS. We summarize them herein after (**Table 1**).

**Table 1:** A list of current developed anti-inflammasome inhibitors potentially applied for COVID-19 treatment.

Classification	Drug name	Reference
**NLRP3 Inhibitors**	NT0793/NT0249	(Mullard, 2019; Freeman and Swartz, 2020)
	β-Hydroxybutyrate (BHB)	(Youm et al., 2015)
	MCC950	(Gordon et al., 2018)
	CY09	(Jiang et al., 2017)
	OLT1177	(Marchetti et al., 2018)
	Tranilast (N-[30, 40 -dimethoxycinnamoyl]-anthranilic acid, TR)	(Huang et al., 2018)
	Oridonin (Ori)	(He et al., 2018)
	Blood POZ-containing gene type-2 (BPO-Z)	(Li et al., 2023)
**GSDMD Inhibitors**	dimethyl fumarate (DMF)	(Vora et al., 2021)
	disulfiram (DSF)	(Vora et al., 2021)
**Immunomodulators**	Anankira	(Gupta, 2020; Khan, 2020)
	CZL-80	(Pan et al., 2022)
	Sennoside A (Sen A)	(Wu et al., 2022)
**siRNA**	experimental trial level for other auto-inflammatory diseases	(Feng et al., 2021; Idris et al., 2021; Mehta et al., 2021)

### NLRP3 inhibitors

#### NT0793/NT0249

Nodthera is a company specialized in development of molecule targeting NLRP3 inflammasome for treatment of multiple diseases such as Parkinson disease, Alzheimer disease (Pipeline - Nodthera, accessed on 17.04.2023). Currently, these molecules have passed preclinical trial and now in phase I (Mullard, 2019; Freeman and Swartz, 2020). Interestingly, it is likely to be used for COVID-19 patient with severe disease as well.

#### β-Hydroxybutyrate

β-Hydroxybutyrate is an endogenous ketone that is able to inhibit NLRP3 through reduction of potassium efflux ASC, and consequently reduce inflammation and IL-1β expression (Youm et al., 2015). It is therefore an optional treatment for COVID-19 leading to severe disease.

#### MCC950

MCC950 is known as NLRP3 inhibitor. It was able to reduce IL-1β production *in vivo* (C57BL/6 female mice) during study for Parkinson’s disease (Gordon et al., 2018).

#### CY09

CY09 a molecule, through binding to the NACHT domain of NLRP3 ATP-binding motif, is able to block inflammasome assembly and activation both *in vitro* and *in vivo* (Jiang et al., 2017).

#### OLT1177

OLT1177 is an active β-sulfonyl nitrile molecule that specifically targets NLRP3 inflammasome. In fact, LPS-stimulated human blood-derived macrophages and monocytes from patients with cryopyrin-associated periodic syndrome (CAPS) treated with OLT1177 presented a decreased level of IL-1β and IL-18. Its mechanism of action is known through inhibition of the binding between the sensor NLRP3 with its adaptor ASC and its interaction with caspase-1 effector (Marchetti et al., 2018).

#### Tranilast

Tranilast is a triptophan analog empirically used as anti-allergic. Later on, studies showed that it exhibits an inhibition activity against NLRP3 inflammasome. In fact, through direct binding to NLRP3 NACHT domain, TR prevents its assembly and oligomerization. The inhibition was efficient both *in vivo* (mouse models of NLRP3 inflammasome-related human diseases) and *in vitro* (mononuclear cells from patients with gout) (Huang et al., 2018).

#### Oridonin

Oridonin is an active compound of *Rabdosia rubescens*, a Chinese traditional medicinal herb. It has been used to treat inflammatory diseases. Currently, its mechanism of action was revealed as a NLRP3 inhibitor through binding to the NLRP3 NACHT domain at cysteine 279. Furthermore, its effects are both preventative and therapeutic during study in mouse models presenting peritonitis, gouty arthritis and type 2 diabetes disease (He et al., 2018).

#### Blood POZ-containing gene type-2

Blood POZ-containing gene type-2 is a cellular molecule involved in growth suppression. Recently it was identified as a negative regulator of NLRP3 inflammasome during SARS-CoV-2 infection. Consequently, it may represent a strategy to be used to inhibit NLRP3 activation which leads to severe COVID-19 syndrome (Li et al., 2023).

Impressively, the NLRP3 inhibitor MCC950 reduced caspase 1 activation and secretion of IL-1β in primary human monocytes infected with SARS-CoV-2 *in vitro* (Rodrigues et al., 2021). Furthermore, several phase II clinical trials are testing direct NLRP3 inhibition in patients with either mild or severe COVID-19, such as NCT04382053 by Novartis (https://clinicaltrials.gov/ct2/history/NCT04382053?V_34=View#StudyPageTop) and NCT04540120 by Olatec Therapeutics (https://www.sciencedirect.com/science/article/pii/B9780323918022000359). These data suggest that NLRP3 inhibition may be a superior strategy against COVID-19 severe symptom.

### GSDMD inhibitors

Gasdermin D is a vital protein during the activation of cell death pathways such as pyroptosis (Burdette et al., 2021). It was able to be highly activated by certain inflammasome proteins (Liu et al., 2016). Therefore, it is an excellent target for the treatment of COVID-19 disease. Dimethyl fumarate (DMF) and disulfiram (DSF) are FDA approved drugs for the treatment of sclerosis and alcoholism, respectively. As they have been identified as GSDMD-inhibitors, their clinical trials are ongoing for the treatment of COVID-19 disease (Vora et al., 2021). In fact, COVID-19 patients under DMF treatment against sclerosis or under DSF against alcohol use disorder showed a self-limiting symptoms (Fillmore et al., 2021; Mantero et al., 2021). Therefore, it is being clinically tested against COVID-19 (trial number NCT04594343) with a treatment dose of 120 mg every 12 hours for 2 days or 240 mg every 12 hours for 8 days. Besides, DSF underwent two clinical trials. The first one was performed in 60 participants with mild disease with a dose of 1000 or 2000mg/day for 5 days (trial number NCT04485130). The second trial was with 200 participants hospitalized and treated with 500mg/day for 14 days (trial number NCT04594343) (Vora et al., 2021).

### Immunomodulators

IL-1β is a part of the cytokine storm that is stimulated by inflammasomes (Martinon et al., 2002). In fact, IL-1 inhibitors have been considered as an excellent candidate for the treatment of SARS-CoV-2-related diseases (Mardi et al., 2021). Most importantly, several immunomodulators are actually commercially available. For instance, Anankira, an immunosuppressive drug used to treat rheumatoid arthritis, have shown considerable survival effects in COVID-19 patients. Its mechanism of action is to inhibit IL-1 by binding to it (Gupta, 2020; Khan, 2020).

Caspases are cysteine-dependent proteases involved in multiple cellular mechanisms including inflammasome activation during COVID-19 infection and thus represent a valuable target (Premeaux et al., 2022). In fact, caspase-1 as an effector of NLRP3 as well as caspase 4 and 8 could be targeted by caspases inhibitors which have been progressively in development (Lee et al., 2018). Indeed, CZL-80 is a caspase-1 inhibitor that had successfully treated mice developing progressive ischemic stroke (PIS) (Pan et al., 2022). Recently, Sennoside A (Sen A) was identified to inhibit caspase-1 and induced the suppression of both NLRP3 and AIM-2 inflammasomes (Wu et al., 2022).

### siRNA

Small interference RNA has always been an attractive strategy to fight against multiple infections including viruses (Saw and Song, 2020). In development of anti-COVID-19 drugs, siRNA has been attempted to interfere with SARS-CoV-2 genomic ssRNA (Idris et al., 2021; Mehta et al., 2021). However, as we know now that the poor prognosis of COVID-19 is not due to the viral infection itself but the activation of inflammasomes leading to tissue damage and organ failure. Thus, targeting a specific type of inflammasome with siRNAs seems to represent a promising strategy. In fact, multiple inflammasomes have already been targeted by siRNA in several auto-inflammatory diseases (Feng et al., 2021).

## Discussion and perspectives

Measures taken towards SARS-CoV-2 pandemic such as restrictions have been removed in almost all countries. However, until now, SARS-CoV-2 is continuing to provoke severe diseases and even death in the world (Chu et al., 2022; Fox et al., 2022). Indeed, finding a powerful and thorough treatment mostly for those leading to severe COVID-19 disease is still an ultimate goal that has to be reached as soon as possible. Moreover, the emergence of new variant of concerns are worrying as they become highly contagious and may lead to a new pandemic wave. Targeting inflammasome, an intracellular protein complex activated in innate immune response following antigen attacks, has been becoming more and more attractive with the increasing investment of many large pharmaceutical companies. Actually, series of inflammasome inhibitors are being tested in the pre- and clinical stages for the treatment of several non-infectious diseases such as CAPS, gouty arthritis, type 2 diabetes etc. For infectious disease like SARS-CoV-2 infection, the cause of cytokine storm is believed due to an excessive immune responses in the organism but not the virus itself (Kaivola et al., 2021). Multiple inflammasomes already exist within the organism and there are multiple alternative ways to produce IL-1 according to the pathogen (virus, bacteria…). Thus, blocking one inflammasome by its inhibitor does not increase the risk of infection and shall not affect the level of innate immune responses. Hence, it represents the best way to fight the infection. In fact, some NLRP3 inhibitors being evaluated under experimental studies have already showed quite satisfactory results (Freeman and Swartz, 2020; Declercq et al., 2022). Besides NLRP3, other types of inflammasomes that were initially known to interact with dsRNA or bacteria such as AIM-2, caspase-4 and -8 were found to play a role in the inflammation responses during COVID-19. Thus, this wide range of molecules represents a broad target spectrum that is likely to be used to develop therapeutics to fight SARS-CoV-2 induced acute lung injury. Therefore, the symptoms may be greatly alleviated through the application of a direct antiviral combined with an inflammasome inhibitor. Nevertheless, further clinical trials have to be carried out to confirm this statement. To ensure the safety of such anti-inflammatory drugs, other non-identified inflammasome that might exist and counteract with SARS-Cov-2 has to be taken in consideration. Moreover, NLRP3 inhibitors does not only suppresses IL-1 but IL-18 as well which is an important regulator for the innate system. This fact may produce an unexpected pathophysiological consequences through a complicated immune network. However, the development of inflammasome inhibitors still hold a promise to treat severe COVID-19 syndrome effectively and eventually reduce mortality.

## Author contributions

KH, RD: conceptualization, literature review, critical and comparative analysis of available information. RD: original manuscript writing and figure preparation. KH: supervision, revision and funding acquisition. YW, DH: comments and suggestion. All authors contributed to the article and approved the submitted version.

## References

[B1] Andargie T. E. Tsuji N. Seifuddin F. Jang M. K. Yuen P. S. Kong H.. (2021). Cell-free DNA maps COVID-19 tissue injury and risk of death and can cause tissue injury. JCI Insight 6 (7), e147610. doi: 10.1172/jci.insight.147610 33651717 PMC8119224

[B2] Awadasseid A. Wu Y. Tanaka Y. Zhang W. (2021). SARS-CoV-2 variants evolved during the early stage of the pandemic and effects of mutations on adaptation in wuhan populations. Int. J. Biol. Sci. 17, 97–106. doi: 10.7150/ijbs.47827 33390836 PMC7757051

[B3] Bauernfried S. Scherr M. J. Pichlmair A. Duderstadt K. E. Hornung V. (2021). Human NLRP1 is a sensor for double-stranded RNA. Sci. (New York N.Y.) 371(6528), eabd0811. doi: 10.1126/science.abd0811 33243852

[B4] Burdette B. E. Esparza A. N. Zhu H. Wang S. (2021). Gasdermin d in pyroptosis. Acta Pharm. Sinica. B 11, 2768–2782. doi: 10.1016/j.apsb.2021.02.006 PMC8463274 34589396

[B5] Cameroni E. Bowen J. E. Rosen L. E. Saliba C. Zepeda S. K. Culap K.. (2022). Broadly neutralizing antibodies overcome SARS-CoV-2 omicron antigenic shift. Nature 602, 664–670. doi: 10.1038/s41586-021-04386-2 35016195 PMC9531318

[B6] Campbell G. R. To R. K. Hanna J. Spector S. A. (2021). SARS-CoV-2, SARS-CoV-1, and HIV-1 derived ssRNA sequences activate the NLRP3 inflammasome in human macrophages through a non-classical pathway. iScience 24, 102295. doi: 10.1016/j.isci.2021.102295 33718825 PMC7939994

[B7] Chu D. T. Vu Ngoc S. M. Vu Thi H. Nguyen Thi Y. V. Ho T. T. Hoang V. T.. (2022). COVID-19 in southeast Asia: current status and perspectives. Bioengineered 13, 3797–3809. doi: 10.1080/21655979.2022.2031417 35081861 PMC8974206

[B8] Colarusso C. Terlizzi M. Maglio A. Molino A. Candia C. Vitale C.. (2022). Activation of the AIM2 receptor in circulating cells of post-COVID-19 patients with signs of lung fibrosis is associated with the release of IL-1α, IFN-α and TGF-β. Front. Immunol. 13. doi: 10.3389/fimmu.2022.934264 PMC9277546 35844548

[B9] Declercq J. De Leeuw E. Lambrecht B. N. (2022). Inflammasomes and IL-1 family cytokines in SARS-CoV-2 infection: from prognostic marker to therapeutic agent. Cytokine 157, 155934. doi: 10.1016/j.cyto.2022.155934 35709568 PMC9170572

[B10] Eltobgy M. M. Zani A. Kenney A. D. Estfanous S. Kim E. Badr A.. (2022). Caspase-4/11 exacerbates disease severity in SARS-CoV-2 infection by promoting inflammation and immunothrombosis. Proc Natl Acad Sci U S A. 119 (21), e2202012119. doi: 10.1073/pnas.2202012119 35588457 PMC9173818

[B11] Feng N. Liang L. Fan M. Du Y. Chen C. Jiang R.. (2021). Treating autoimmune inflammatory diseases with an siERN1-nanoprodrug that mediates macrophage polarization and blocks toll-like receptor signaling. ACS nano 15, 15874–15891. doi: 10.1021/acsnano.1c03726 34586802

[B12] Fillmore N. Bell S. Shen C. Nguyen V. La J. Dubreuil M.. (2021). Disulfiram use is associated with lower risk of COVID-19: a retrospective cohort study. PloS One 16, e0259061. doi: 10.1371/journal.pone.0259061 34710137 PMC8553043

[B13] Fox T. Geppert J. Dinnes J. Scandrett K. Bigio J. Sulis G.. (2022). Antibody tests for identification of current and past infection with SARS-CoV-2. Cochrane Database systematic Rev. 11, CD013652. doi: 10.1002/14651858.CD013652.pub2 PMC9671206 36394900

[B14] Freeman T. L. Swartz T. H. (2020). Targeting the NLRP3 inflammasome in severe COVID-19. Front. Immunol. 11. doi: 10.3389/fimmu.2020.01518 PMC7324760 32655582

[B15] Gordon R. Albornoz E. A. Christie D. C. Langley M. R. Kumar V. Mantovani S.. (2018). Inflammasome inhibition prevents α-synuclein pathology and dopaminergic neurodegeneration in mice. Sci. Trans. Med. 10 (465), eaah4066. doi: 10.1126/scitranslmed.aah4066 PMC6483075 30381407

[B16] Gupta R. (2020). Anakinra: a silver lining in COVID-19? Crit. Care (London England) 24, 598. doi: 10.1186/s13054-020-03312-8 PMC7537962 33023646

[B17] Hadj Hassine I. (2022). Covid-19 vaccines and variants of concern: a review. Rev. Med. Virol. 32, e2313. doi: 10.1002/rmv.2313 34755408 PMC8646685

[B18] Harvey W. T. Carabelli A. M. Jackson B. Gupta R. K. Thomson E. C. Harrison E. M.. (2021). SARS-CoV-2 variants, spike mutations and immune escape. Nat. Rev. Microbiol. 19, 409–424. doi: 10.1038/s41579-021-00573-0 34075212 PMC8167834

[B19] He L. Ding Y. Zhang Q. Che X. He Y. Shen H.. (2006). Expression of elevated levels of pro-inflammatory cytokines in SARS-CoV-infected ACE2+ cells in SARS patients: relation to the acute lung injury and pathogenesis of SARS. J. Pathol. 210, 288–297. doi: 10.1002/path.2067 17031779 PMC7167655

[B20] He H. Jiang H. Chen Y. Ye J. Wang A. Wang C.. (2018). Oridonin is a covalent NLRP3 inhibitor with strong anti-inflammasome activity. Nat. Commun. 9, 2550. doi: 10.1038/s41467-018-04947-6 29959312 PMC6026158

[B21] Huang Y. Jiang H. Chen Y. Wang X. Yang Y. Tao J.. (2018). Tranilast directly targets NLRP3 to treat inflammasome-driven diseases. EMBO Mol. Med. 10 (4), e8689. doi: 10.15252/emmm.201708689 29531021 PMC5887903

[B22] Huang C. Wang Y. Li X. Ren L. Zhao J. Hu Y.. (2020). Clinical features of patients infected with 2019 novel coronavirus in wuhan, China. Lancet (London England) 395, 497–506. doi: 10.1016/s0140-6736(20)30183-5 31986264 PMC7159299

[B23] Idris A. Davis A. Supramaniam A. Acharya D. Kelly G. Tayyar Y.. (2021). A SARS-CoV-2 targeted siRNA-nanoparticle therapy for COVID-19. Mol. Ther. J. Am. Soc. Gene Ther. 29, 2219–2226. doi: 10.1016/j.ymthe.2021.05.004 PMC8118699 33992805

[B24] Jiang H. He H. Chen Y. Huang W. Cheng J. Ye J.. (2017). Identification of a selective and direct NLRP3 inhibitor to treat inflammatory disorders. J. Exp. Med. 214, 3219–3238. doi: 10.1084/jem.20171419 29021150 PMC5679172

[B25] Junqueira C. Crespo Â. Ranjbar S. Lewandrowski M. Ingber J. de Lacerda L. B.. (2021). SARS-CoV-2 infects blood monocytes to activate NLRP3 and AIM2 inflammasomes, pyroptosis and cytokine release. Res. Square. rs.3.rs–153628. doi: 10.21203/rs.3.rs-153628/v1

[B26] Kaivola J. Nyman T. A. Matikainen S. (2021). Inflammasomes and SARS-CoV-2 infection. Viruses 13 (12), 2513. doi: 10.3390/v13122513 34960782 PMC8706865

[B27] Khan N. A. (2020). Anakinra for severe forms of COVID-19. Lancet Rheumatol. 2, e586–e587. doi: 10.1016/s2665-9913(20)30273-3 PMC7413653 32838320

[B28] Kim D. Lee J. Y. Yang J. S. Kim J. W. Kim V. N. Chang H. (2020). The architecture of SARS-CoV-2 transcriptome. Cell 181, 914–921.e910. doi: 10.1016/j.cell.2020.04.011 32330414 PMC7179501

[B29] Kruse R. L. (2020). Therapeutic strategies in an outbreak scenario to treat the novel coronavirus originating in wuhan, China. F1000Research 9, 72. doi: 10.12688/f1000research.22211.2 32117569 PMC7029759

[B30] Kumar S. Thambiraja T. S. Karuppanan K. Subramaniam G. (2022). Omicron and delta variant of SARS-CoV-2: a comparative computational study of spike protein. J. Med. Virol. 94, 1641–1649. doi: 10.1002/jmv.27526 34914115

[B31] Lee S. Channappanavar R. Kanneganti T. D. (2020). Coronaviruses: innate immunity, inflammasome activation, inflammatory cell death, and cytokines. Trends Immunol. 41, 1083–1099. doi: 10.1016/j.it.2020.10.005 33153908 PMC7561287

[B32] Lee H. Shin E. A. Lee J. H. Ahn D. Kim C. G. Kim J. H.. (2018). Caspase inhibitors: a review of recently patented compounds (2013-2015). Expert Opin. Ther. patents 28, 47–59. doi: 10.1080/13543776.2017.1378426 28885866

[B33] Li J. Lin H. Fan T. Huang L. Zhang X. Tai Y.. (2023). BPOZ-2 is a negative regulator of the NLPR3 inflammasome contributing to SARS-CoV-2-induced hyperinflammation. Front. Cell. infection Microbiol. 13. doi: 10.3389/fcimb.2023.1134511 PMC10019892 36936774

[B34] Li S. Zhang Y. Guan Z. Li H. Ye M. Chen X.. (2020). SARS-CoV-2 triggers inflammatory responses and cell death through caspase-8 activation. Signal transduction targeted Ther. 5, 235. doi: 10.1038/s41392-020-00334-0 PMC7545816 33037188

[B35] Liu X. Zhang Z. Ruan J. Pan Y. Magupalli V. G. Wu H.. (2016). Inflammasome-activated gasdermin d causes pyroptosis by forming membrane pores. Nature 535, 153–158. doi: 10.1038/nature18629 27383986 PMC5539988

[B36] Lugrin J. Martinon F. (2018). The AIM2 inflammasome: sensor of pathogens and cellular perturbations. Immunol. Rev. 281, 99–114. doi: 10.1111/imr.12618 29247998

[B37] Maes M. Tedesco Junior W. L. D. Lozovoy M. A. B. Mori M. T. E. Danelli T. Almeida E. R. D.. (2022). In COVID-19, NLRP3 inflammasome genetic variants are associated with critical disease and these effects are partly mediated by the sickness symptom complex: a nomothetic network approach. Mol. Psychiatry 27, 1945–1955. doi: 10.1038/s41380-021-01431-4 35022530 PMC8752583

[B38] Majumder J. Minko T. (2021). Recent developments on therapeutic and diagnostic approaches for COVID-19. AAPS J. 23, 14. doi: 10.1208/s12248-020-00532-2 33400058 PMC7784226

[B39] Mantero V. Abate L. Basilico P. Balgera R. Salmaggi A. Nourbakhsh B.. (2021). COVID-19 in dimethyl fumarate-treated patients with multiple sclerosis. J. Neurol. 268, 2023–2025. doi: 10.1007/s00415-020-10015-1 32588182 PMC7314911

[B40] Marchetti C. Swartzwelter B. Gamboni F. Neff C. P. Richter K. Azam T.. (2018). OLT1177, a β-sulfonyl nitrile compound, safe in humans, inhibits the NLRP3 inflammasome and reverses the metabolic cost of inflammation. Proc Natl Acad Sci U S A. 115 (7), E1530–E1539. doi: 10.1073/pnas.1716095115 29378952 PMC5816172

[B41] Mardi A. Meidaninikjeh S. Nikfarjam S. Majidi Zolbanin N. Jafari R. (2021). Interleukin-1 in COVID-19 infection: immunopathogenesis and possible therapeutic perspective. Viral Immunol. 34, 679–688. doi: 10.1089/vim.2021.0071 34882013

[B42] Martinon F. Burns K. Tschopp J. (2002). The inflammasome: a molecular platform triggering activation of inflammatory caspases and processing of proIL-beta. Mol. Cell 10, 417–426. doi: 10.1016/s1097-2765(02)00599-3 12191486

[B43] Mehta A. Michler T. Merkel O. M. (2021). siRNA therapeutics against respiratory viral infections-what have we learned for potential COVID-19 therapies? Advanced healthcare materials 10, e2001650. doi: 10.1002/adhm.202001650 33506607 PMC7995229

[B44] Mullard A. (2019). NLRP3 inhibitors stoke anti-inflammatory ambitions. Nat. Rev. Drug Discovery 18, 405–407. doi: 10.1038/d41573-019-00086-9 31160775

[B45] Pan P. Shen M. Yu Z. Ge W. Chen K. Tian M.. (2021). SARS-CoV-2 n protein promotes NLRP3 inflammasome activation to induce hyperinflammation. Nat. Commun. 12, 4664. doi: 10.1038/s41467-021-25015-6 34341353 PMC8329225

[B46] Pan L. Tang W. D. Wang K. Fang Q. F. Liu M. R. Wu Z. X.. (2022). Novel caspase-1 inhibitor CZL80 improves neurological function in mice after progressive ischemic stroke within a long therapeutic time-window. Acta pharmacologica Sin. 43, 2817–2827. doi: 10.1038/s41401-022-00913-7 PMC9622895 35501362

[B47] Peng Y. Mentzer A. J. Liu G. Yao X. Yin Z. Dong D.. (2020). Broad and strong memory CD4(+) and CD8(+) T cells induced by SARS-CoV-2 in UK convalescent individuals following COVID-19. Nat. Immunol. 21, 1336–1345. doi: 10.1038/s41590-020-0782-6 32887977 PMC7611020

[B48] Planès R. Pinilla M. Santoni K. Hessel A. Passemar C. Lay K.. (2022). Human NLRP1 is a sensor of pathogenic coronavirus 3CL proteases in lung epithelial cells. Mol. Cell 82, 2385–2400.e2389. doi: 10.1016/j.molcel.2022.04.033 35594856 PMC9108100

[B49] Premeaux T. A. Yeung S. T. Bukhari Z. Bowler S. Alpan O. Gupta R.. (2022). Emerging insights on caspases in COVID-19 pathogenesis, sequelae, and directed therapies. Front. Immunol. 13. doi: 10.3389/fimmu.2022.842740 PMC8899608 35265086

[B50] Robinson P. C. Liew D. F. L. Tanner H. L. Grainger J. R. Dwek R. A. Reisler R. B.. (2022). COVID-19 therapeutics: challenges and directions for the future. Proc Natl Acad Sci U S A. 119 (15), e2119893119. doi: 10.1073/pnas.2119893119 35385354 PMC9169797

[B51] Robinson K. S. Teo D. E. T. Tan K. S. Toh G. A. Ong H. H. Lim C. K.. (2020). Enteroviral 3C protease activates the human NLRP1 inflammasome in airway epithelia. Sci. (New York N.Y.) 370 (6521), eaay2002. doi: 10.1126/science.aay2002 33093214

[B52] Rodrigues T. S. de Sá K. S. G. Ishimoto A. Y. Becerra A. Oliveira S. Almeida L.. (2021). Inflammasomes are activated in response to SARS-CoV-2 infection and are associated with COVID-19 severity in patients. J. Exp. Med. 218 (3), e20201707. doi: 10.1084/jem.20201707 33231615 PMC7684031

[B53] Saw P. E. Song E. W. (2020). siRNA therapeutics: a clinical reality. Sci. China. Life Sci. 63, 485–500. doi: 10.1007/s11427-018-9438-y 31054052

[B54] Scovino A. M. Dahab E. C. Vieira G. F. Freire-de-Lima L. Freire-de-Lima C. G. Morrot A. (2022). SARS-CoV-2's variants of concern: a brief characterization. Front. Immunol. 13. doi: 10.3389/fimmu.2022.834098 PMC9361785 35958548

[B55] Sola I. Almazán F. Zúñiga S. Enjuanes L. (2015). Continuous and discontinuous RNA synthesis in coronaviruses. Annu. Rev. Virol. 2, 265–288. doi: 10.1146/annurev-virology-100114-055218 26958916 PMC6025776

[B56] Viganò E. Diamond C. E. Spreafico R. Balachander A. Sobota R. M. Mortellaro A. (2015). Human caspase-4 and caspase-5 regulate the one-step non-canonical inflammasome activation in monocytes. Nat. Commun. 6, 8761. doi: 10.1038/ncomms9761 26508369 PMC4640152

[B57] Vora S. M. Lieberman J. Wu H. (2021). Inflammasome activation at the crux of severe COVID-19. Nat. Rev. Immunol. 21, 694–703. doi: 10.1038/s41577-021-00588-x 34373622 PMC8351223

[B58] Wang D. Hu B. Hu C. Zhu F. Liu X. Zhang J.. (2020). Clinical characteristics of 138 hospitalized patients with 2019 novel coronavirus-infected pneumonia in wuhan, China. Jama 323, 1061–1069. doi: 10.1001/jama.2020.1585 32031570 PMC7042881

[B59] Wu J. Lan Y. Shi X. Huang W. Li S. Zhang J.. (2022). Sennoside a is a novel inhibitor targeting caspase-1. Food Funct. 13, 9782–9795. doi: 10.1039/d2fo01730j 36097956

[B60] Xu H. Akinyemi I. A. Chitre S. A. Loeb J. C. Lednicky J. A. McIntosh M. T.. (2022). SARS-CoV-2 viroporin encoded by ORF3a triggers the NLRP3 inflammatory pathway. Virology 568, 13–22. doi: 10.1016/j.virol.2022.01.003 35066302 PMC8762580

[B61] Yap J. K. Y. Moriyama M. Iwasaki A. (2020). Inflammasomes and pyroptosis as therapeutic targets for COVID-19. J. Immunol. (Baltimore Md. 1950) 205, 307–312. doi: 10.4049/jimmunol.2000513 PMC7343621 32493814

[B62] Youm Y. H. Nguyen K. Y. Grant R. W. Goldberg E. L. Bodogai M. Kim D.. (2015). The ketone metabolite β-hydroxybutyrate blocks NLRP3 inflammasome-mediated inflammatory disease. Nat. Med. 21, 263–269. doi: 10.1038/nm.3804 25686106 PMC4352123

[B63] Zeng B. Gao L. Zhou Q. Yu K. Sun F. (2022). Effectiveness of COVID-19 vaccines against SARS-CoV-2 variants of concern: a systematic review and meta-analysis. BMC Med. 20, 200. doi: 10.1186/s12916-022-02397-y 35606843 PMC9126103

[B64] Zhao N. Di B. Xu L. L. (2021). The NLRP3 inflammasome and COVID-19: activation, pathogenesis and therapeutic strategies. Cytokine Growth factor Rev. 61, 2–15. doi: 10.1016/j.cytogfr.2021.06.002 34183243 PMC8233448

